# Supplementary material for: Protonated Clusters of Neon and Krypton
Source: J Am Soc Mass Spectrom. 2019 Oct 24;30(12):2632–6. doi: 10.1007/s13361-019-02329-w (PMC6914732; doi:10.1007/s13361-019-02329-w)
Supplement: Supplementary file 1 — (PDF 74 kb) [file 13361_2019_2329_MOESM1_ESM.pdf]

Electronic supplementary material for "Protonated Clusters of Neon and Krypton"

Michael Gatchell,[1,2,*] Paul Martini,[1] Arne Schiller,[1] and Paul Scheier[1]

[1] *Institut für Ionenphysik und Angewandte Physik, Universität Innsbruck, Technikerstr. 25, A-6020 Innsbruck, Austria*

[2] *Department of Physics, Stockholm University, 106 91 Stockholm, Sweden*

The electronic energy and coordinates of the structures shown in Figures 4 and 5 of the main paper calculated at MP2(Full)/def2-SVPP level. The energies are given in units of hartree and do not contain zero-point energy corrections. The spatial cartesian coordinates are given in units of Ångström (10^{-10} m).

Ne_7H^+

E = -900.04306676

Ne	1.21826	0.32708	-1.18406
Ne	-1.07808	0.92938	-0.03343
Ne	0.42257	-1.17079	0.51126
H	0.82126	-0.42204	-0.33626
Ne	1.32321	1.17375	1.31298
Ne	-0.85517	-1.18513	-1.79530
Ne	1.68206	-2.24493	-1.54197
Ne	3.02861	-0.78487	0.37733

Ne_{19}

E = -2442.68923204

Ne	-0.86352	1.30447	-3.56534
Ne	0.85870	-1.30503	3.56597
Ne	1.25091	2.37030	-2.04366
Ne	1.43219	-0.24058	-3.04261
Ne	-1.39125	2.80704	-1.24007
Ne	-1.09894	-1.41261	-2.85321
Ne	-2.84335	0.47348	-1.74323
Ne	2.30983	0.77059	2.33294
Ne	2.48966	-1.83956	1.33425
Ne	-0.33484	1.20739	3.12552
Ne	-0.04170	-3.00981	1.51510
Ne	-1.78614	-1.12336	2.62307
Ne	2.30225	0.32455	-0.43848
Ne	0.57000	2.19140	0.66436
Ne	0.85792	-1.99141	-0.93611
Ne	-1.94782	1.02889	0.84572
Ne	-1.76743	-1.55605	-0.14207
Ne	0.28475	-0.42725	1.16610
Ne	-0.28123	0.42755	-1.16824

Ne_{19}^{+}

E = -2441.97237061

Ne	-0.71622	1.09465	-2.99904
Ne	0.73379	-1.10261	3.00834
Ne	1.29052	2.30639	-1.84990
Ne	1.47732	-0.31379	-2.84911
Ne	-1.34852	2.73848	-1.07319
Ne	-1.06166	-1.47313	-2.68290
Ne	-2.78518	0.40800	-1.55978
Ne	2.25289	0.83406	2.13931
Ne	2.43307	-1.76272	1.13908
Ne	-0.37192	1.26006	2.95191
Ne	-0.08642	-2.93626	1.34274
Ne	-1.81350	-1.05640	2.43365
Ne	2.42325	0.35245	-0.46321
Ne	0.57162	2.31173	0.70705
Ne	0.87679	-2.10982	-0.98707
Ne	-2.03602	1.09505	0.89284
Ne	-1.85275	-1.64147	-0.15572
Ne	0.20289	-0.29881	0.81175
Ne	-0.18996	0.29414	-0.80675

$Ne_{19}H^{+}$

E = -2442.80098036

Ne	-0.84626	1.26686	-3.44749
Ne	0.84298	-1.26769	3.45640
Ne	1.23807	2.32902	-1.98874
Ne	1.41371	-0.24069	-2.97374
Ne	-1.37145	2.76022	-1.18960
Ne	-1.08334	-1.39973	-2.78962
Ne	-2.80576	0.45583	-1.68426
Ne	2.27898	0.76730	2.27167
Ne	2.45559	-1.80493	1.28669
Ne	-0.32830	1.19814	3.06709
Ne	-0.04242	-2.96348	1.46943
Ne	-1.76283	-1.10897	2.57148
Ne	2.28933	0.32497	-0.43561
Ne	0.56603	2.18598	0.66800
Ne	0.85197	-1.98550	-0.93222
Ne	-1.94130	1.02443	0.85613
Ne	-1.76253	-1.55581	-0.13681
Ne	0.26568	-0.40114	1.09522
Ne	-0.26807	0.40027	-1.08760

H	-0.00174	-0.00020	0.00409

Kr_{19}

E = -52286.73506904

Kr	-1.28276	1.93658	-5.28984
Kr	1.27548	-1.93394	5.29244
Kr	1.86908	3.51923	-3.02955
Kr	2.13677	-0.35828	-4.51310
Kr	-2.06325	4.17224	-1.83153
Kr	-1.62962	-2.10853	-4.23340
Kr	-4.22766	0.69852	-2.58795
Kr	3.43447	1.14660	3.45472
Kr	3.70361	-2.73014	1.97017
Kr	-0.49848	1.80489	4.63921
Kr	-0.06391	-4.47472	2.23559
Kr	-2.65559	-1.67547	3.89746
Kr	3.42211	0.48602	-0.65136
Kr	0.84722	3.26547	0.98957
Kr	1.27650	-2.96438	-1.39424
Kr	-2.90243	1.52947	1.26278
Kr	-2.63896	-2.31556	-0.21029
Kr	0.41661	-0.63131	1.72639
Kr	-0.41919	0.63328	-1.72706

Kr_{19}^{+}

E = -52286.29111397

Kr	-1.01132	1.53808	-4.21462
Kr	1.04287	-1.57318	4.29265
Kr	1.98644	3.50473	-2.77144
Kr	2.27004	-0.50208	-4.30070
Kr	-2.04241	4.16315	-1.57101
Kr	-1.59755	-2.27698	-4.03516
Kr	-4.25150	0.60375	-2.32299
Kr	3.41002	1.27950	3.19238
Kr	3.68287	-2.65263	1.68158
Kr	-0.57678	1.93360	4.41814
Kr	-0.14480	-4.43065	1.97734
Kr	-2.76952	-1.58625	3.63851
Kr	3.52811	0.51262	-0.68515
Kr	0.84958	3.37317	1.02179
Kr	1.29394	-3.06610	-1.44350
Kr	-2.98123	1.59357	1.29629

Kr	-2.71283	-2.38962	-0.23447
Kr	0.32817	-0.49095	1.33735
Kr	-0.30412	0.46623	-1.27699

$Kr_{19}H^+$

E = -52286.92684457

Kr	-1.19337	1.80358	-4.92748
Kr	1.19166	-1.80426	4.93327
Kr	1.85826	3.43912	-2.90743
Kr	2.12115	-0.37963	-4.36871
Kr	-2.01610	4.08329	-1.73348
Kr	-1.58855	-2.09876	-4.09971
Kr	-4.14801	0.65862	-2.47068
Kr	3.36888	1.15671	3.33231
Kr	3.63166	-2.66373	1.87132
Kr	-0.50741	1.80230	4.50510
Kr	-0.07970	-4.38300	2.13937
Kr	-2.64065	-1.62375	3.76740
Kr	3.40119	0.48050	-0.64274
Kr	0.83918	3.24839	0.98978
Kr	1.26606	-2.95026	-1.38154
Kr	-2.88718	1.52591	1.26096
Kr	-2.62058	-2.30898	-0.20670
Kr	0.37187	-0.56695	1.55482
Kr	-0.37742	0.56646	-1.54287
H	-0.00263	-0.00070	0.00749
